# Accounting for Proportion Congruency Effects in the Stroop Task in a Confounded Setup: Retrieval of Stimulus-Response Episodes Explains it All

**DOI:** 10.5334/joc.232

**Published:** 2022-06-29

**Authors:** Klaus Rothermund, Nathalie Gollnick, Carina G. Giesen

**Affiliations:** 1 Friedrich Schiller University Jena, Jena, Germany

**Keywords:** Stroop task, proportion congruency effect, stimulus-response bindings, cognitive control, contingency learning, episodic response retrieval, episodic retrieval of control states, law of recency

## Abstract

Proportion congruency (PC) effects on the strength of distractor interference were investigated in a high-powered (n = 109), pre-registered experiment in which participants had to identify the ink color of color words. Replicating the standard PC effect, Stroop interference was larger in blocks comprising mostly congruent word-color combinations, compared to blocks comprising mostly incongruent trials. These block-level differences in the strength of the Stroop effect were eliminated after controlling for (a) the congruency of the most recent episode in which the current word had been presented (“episodic retrieval of control states”), and also after controlling for (b) the response relation of this episode and the current trial (“episodic response retrieval”). Controlling for the congruency in trial n-1 (congruency sequence effect, CSE), irrespective of word relation did not eliminate the PC effect, nor did controlling for immediate exact and partial repetitions. When predicting PC effects simultaneously by both types of episodic retrieval processes, only episodic response retrieval explained the effect. Our findings attest to the importance of episodic response retrieval processes in explaining the PC effect in Stroop-like tasks in a confounded setup where different processes compete with each, and they speak against explanations in terms of a global adjustment of cognitive control settings or contingency learning under these conditions. The results further support the assumption that the most recent episode in which a stimulus had occurred is crucial for responding in the current trial (the “law of recency”; Giesen et al., 2020).

The Stroop paradigm (Stroop, 1935) is one of the most popular tasks in Cognitive Psychology, and has been extensively used to study how the human mind deals with conflicting information (for reviews, see Dyer, 1973; MacLeod, 1991). In its original version, the task requires identifying the print color of color words, showing robust interference in responding to incongruent color-word pairs (e.g., BLUE printed in red ink) compared to congruent color-word pairs (e.g., BLUE printed in blue ink). Structurally, the Stroop task has been categorized as a stimulus-response compatibility task in which the irrelevant stimulus dimension (i.e., the word) can be either compatible or incompatible with the relevant stimulus dimension (i.e., the color) and the to-be-executed response (Kornblum et al., 1990). This structural analysis revealed that superficially different paradigms like Evaluative Priming or the Flanker task are in fact structurally similar to the Stroop paradigm (De Houwer, 2003; Klauer et al., 2003).

More recently, research with Stroop-like tasks mostly focused on the analysis of context effects. In a groundbreaking study, Logan and Zbrodoff (1979) demonstrated that the strength of the Stroop effect depends on the proportion of congruent and incongruent trials (proportion congruency [PC] effect). They found a substantially reduced Stroop effect in blocks with a high frequency of incongruent trials compared with blocks with mostly congruent trials. Such a reduction in the strength of Stroop interference has also been observed for specific words that were mostly presented in an incongruent color compared to words that were mostly presented in a congruent color (Jacoby et al., 2003).

To explicate these effects, different explanatory accounts have been proposed (see Figure 1 for an illustration of the predictions of the various accounts). A first set of explanations focused on *cognitive control processes* that put more or less attentional weight on the irrelevant stimulus dimension, depending on whether this dimension was helpful (large proportion of congruent combinations) or detrimental (large proportion of incongruent combinations) for performance on a majority of trials. These cognitive control processes can be assumed to operate on a global scale (based on the proportion of congruent trials in a block; e.g., Klauer et al., 2003; Logan & Zbrodoff, 1979) or on a local scale (based on the immediately preceding trial, the so-called congruency sequence effect [CSE]; Gratton et al., 1992). According to a global cognitive control account, a block with mostly congruent color-word combinations will trigger an open mode of processing, in which the irrelevant stimulus dimension has a strong influence on responding, resulting in a strong Stroop effect; in turn, a block with mostly incongruent color-word combinations will trigger a focused processing mode, in which the irrelevant stimulus dimension has a weak influence on responding, resulting in a weak Stroop effect (Botvinick et al., 2001; Egner, 2014; see Figure 1A). A local cognitive control account would predict strong (weak) Stroop effects after immediately preceding trials that were congruent (incongruent) (see Figure 1B; note that block-wise congruency is typically not regarded in analyses of trial-wise adjustment of cognitive control; however, to highlight the predictions of this account as an explanatory account for [block-wise] PC effects, block was added as an additional factor in Figure 1B).

**Figure 1:**
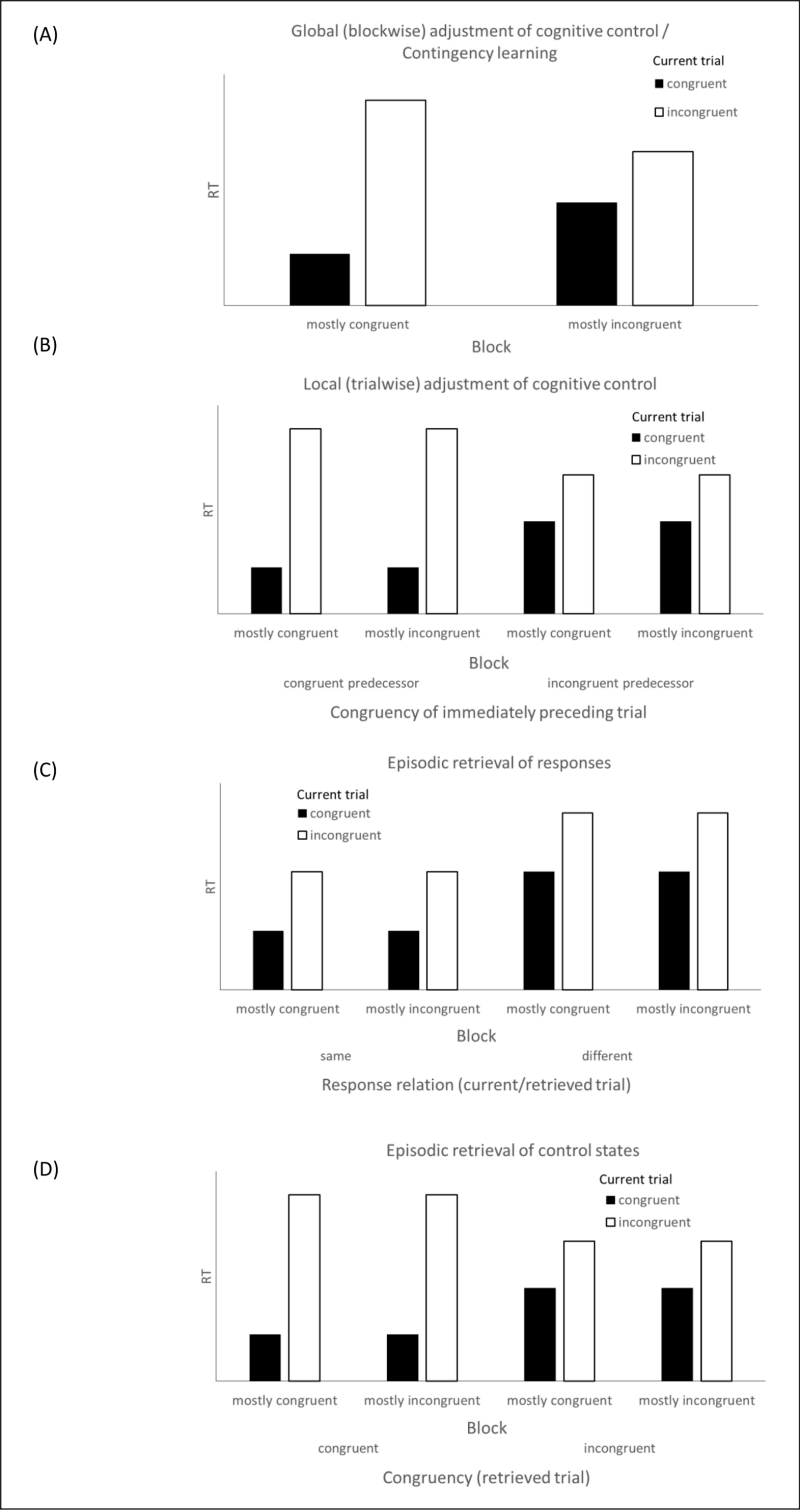
Proportion congruency effects in the Stroop task as predicted by different accounts.

Additionally, cognitive control can be assumed to operate in a general mode (independent of specific stimuli; Botvinick et al., 2001; Logan & Zbrodoff, 1979) or in an item-specific fashion (triggered by specific stimuli; Jacoby et al., 2003). For a task variant in which proportion congruency is manipulated at the block level as it was done in the current study, with proportion congruency being manipulated consistently for all items of a block, both variants of the cognitive control account lead to the same prediction.

According to a second account, PC effects in the Stroop task can be explained in terms of *contingency learning* (Schmidt & Besner, 2008). Rather than shifting attention towards or away from an irrelevant stimulus dimension, the irrelevant stimulus dimension, that is, the word meaning (which we will refer to as *distractor*) becomes associated to those responses with which a specific word has systematically been paired during the task. Presenting the word BLUE in blue ink for most of the trials (i.e., in a mostly congruent condition) establishes a contingency between this word and the blue response. This association produces faster responses on congruent trials, but leads to interference on incongruent trials, which explains why the Stroop effect is stronger for mostly congruent pairings (see Figure 1A). Such an account explains both general but also item-specific PC effects (Schmidt & Besner, 2008). In a study like ours, in which proportion congruency is manipulated homogeneously for all items within a block, the prediction of the contingency learning account is indistinguishable from the prediction of a global cognitive control adaptation account.

A third group of explanations focuses on episodic stimulus-response retrieval processes (see Frings et al., 2020, for a recent review). Episodic retrieval accounts assume that a distractor word retrieves the most recent episode in which the word has been presented previously, and retrieves the response information from that episode (this principle of retrieving the last, most recent episode has been labelled as the “law of recency”; Giesen et al., 2020). Episodic stimulus-based response (SR) retrieval has already been used to explain a variety of effects in different cognitive psychology paradigms (e.g., Negative Priming effects, Rothermund et al., 2005, see also Mayr & Buchner, 2006; task switching costs, Koch et al., 2018; conflict adaptation effects, Mayr et al., 2003). Most recently, episodic SR retrieval has also been used to explain effects of SR contingency learning (Giesen et al., 2020; Schmidt et al., 2020). Similar to the contingency learning account, episodic retrieval accounts attribute PC effects in the Stroop task to stimulus-response learning. In contrast to contingency learning accounts, which assume that contingencies are learned and applied in each trial, the core assumption of episodic retrieval accounts is that contingency effects are due to a specific, stimulus-based retrieval of the last episode in which the current stimulus occurred. It is always the very last episode in which a stimulus was presented that is retrieved, which is most often (but not always) congruent in a high proportion congruent condition, and most often (but not always) incongruent in a high proportion incongruent condition. In the comparatively few cases in which an infrequent combination is retrieved from the last episode (i.e., when the last episode was incongruent in a mostly congruent condition, or congruent in a mostly incongruent condition), the episodic retrieval account makes a prediction that is opposite to the contingency learning account. That is, episodic response retrieval predicts facilitation (interference) if the retrieved response matches (conflicts with) the response that is required on the current trial (see Figure 1C) – even if the retrieved response is infrequent in the current context – whereas the contingency learning account would predict facilitation or interference solely based on what is the more frequent response in the current context. Recent studies revealed that episodic retrieval is a better predictor of performance than contingency learning, and explains most if not all of the contingency learning effect (Giesen et al., 2020; Schmidt et al., 2020; but see Xu & Mordkoff, 2020).

Another explanatory variant combines the ideas of retrieval with cognitive control accounts under the label of “context-dependent retrieval of control states” (Dignath et al., 2019; Dignath & Kiesel, 2021; see also Egner, 2014; Crump & Milliken, 2009; Crump et al., 2006). According to the standard version of such an account, contexts can become associated with the average control states that are typically required in the respective context; that is, contexts with a high frequency of incongruent (congruent) trials become associated with high (low) levels of control. For a standard PC design, where proportion congruency is manipulated in a simple, block-wise fashion, predictions of such a context-dependent retrieval account are indistinguishable from the predictions of accounts in terms of a global adjustment of cognitive control or from a contingency learning account.

The idea of a context-dependent retrieval of control states, however, can also be translated into an episodic, stimulus-based retrieval account in which the distractor functions as a context. According to such an episodic, instance-based variant of the retrieval of control states account, the distractor of the current trial retrieves the control state of the most recent episode in which the current distractor occurred. Such an account can also explain PC effects: In a mostly congruent block, what is retrieved by a distractor word from the last episode is typically (since most trials are congruent) a relaxed state of control which fosters processing also of the irrelevant dimension. Retrieving such an open mode of processing will lead to fast responses on congruent, but slow or erroneous responses on incongruent trials (see Figure 1D). In a mostly incongruent condition, however, the distractor will typically retrieve a rigid control state that is focused on the relevant information. Retrieving such a control state will reduce the influence of the irrelevant stimulus dimension in the current trial, and will lead to a reduction of the Stroop interference effect (see Figure 1D).

The specific influence of these different processes on the PC effect is hard to determine in a standard setup, since under these conditions, the different processes show a large degree of overlap. Blocks consisting of a high proportion of congruent trials may produce large Stroop effects due to global and/or local adjustments of cognitive control, increasing the attentional weight of the distractor words. The same blocks, however, are also characterized by a positive word-color contingency, which can explain why responses are much faster on congruent trials that occur more frequently in those blocks. Furthermore, episodic retrieval processes in mostly congruent blocks are biased towards the retrieval of matching (mismatching) responses for congruent (incongruent) trials, and/or they will retrieve control settings from previous congruent trials in which distractor words were given large weights. Episodic response retrieval and retrieval of control states will thus also produce larger Stroop effects in mostly congruent blocks. Due to the confounding of these processes, it is notoriously hard to disentangle their unique influence on the PC effect.

Multiple experimental studies have been conducted that tested the explanatory potential of some of these various accounts for explaining proportion congruency effects. Most of the previous research focused on the comparison of cognitive control and contingency learning accounts. These studies produced evidence for both accounts, showing that differences in item-based contingencies explained PC effects independently of the overall proportion congruency (for reviews, see Schmidt, 2013a; 2019), but also showing that differences in the global proportion congruency had an influence on items for which the contingencies between word and color were fixed (e.g., Spinelli & Lupker, 2021).

The contribution of episodic retrieval processes on the PC effect, on the other hand, has not yet been investigated systematically (for a recent exception, see Jiménez et al., 2021). In particular, no systematic study has yet been conducted that tested influences of different types of episodic retrieval (retrieval of responses, retrieval of control states) simultaneously, allowing for a competition between these different accounts. In addition, rather than having episodic retrieval accounts compete with the other (control-based, contingency-based) accounts, previous studies rather aimed at eliminating influences of stimulus-based retrieval by avoiding stimulus overlap or by excluding immediate stimulus repetitions from the task or from the computation of effects (cf. Braem et al., 2019). On the one hand, such an experimental strategy of disentangling the influence of different processes by creating conditions under which only one process is operative while all other processes are eliminated is optimal for testing whether a specific process actually exists. On the other hand, these experimental strategies sometimes also have limitations, some of which apply in particular to the study of PC effects in Stroop-like tasks:

1. Attempts to eliminate complete or partial repetitions often do not fully eliminate stimulus-based retrieval processes, since only immediate repetitions were excluded (e.g., Jiménez et al., 2021; Xu & Mordkoff, 2021), which is a suboptimal strategy not just for an analysis of episodic retrieval processes (Aben et al., 2017).
2. It may sometimes be difficult – if not impossible – to fully eliminate all processes but one experimentally, since many of these processes are triggered simultaneously by similar situations. For instance, whenever a specific episode is retrieved from memory, such a retrieval could in principle involve both the response and the control state that were tied to this episode. A recent study by Cochrane and Pratt (2022) also demonstrated an influence of retrieval processes in the item-specific PC effect, which was formerly assumed to reflect effects of pure long-term associative learning.
3. Experiments that eliminate the influence of many processes do not allow for a direct comparison of the contribution of the different processes (e.g., episodic retrieval-based processes in comparison to control-based adjustments and contingency learning) under conditions where these different processes compete with each other. Such a comparison under confounded conditions, however, may sometimes be the core research question: For instance, testing whether abstract, associative contingency learning influences responding over and above episodic, instance-based retrieval processes can only be achieved in a situation in which both effects are operative (e.g., Giesen et al., 2020; Jiménez et al., 2021; Schmidt et al., 2020; Xu & Mordkoff, 2021).
4. Relatedly, creating conditions that eliminate the influence of all processes except one often result in artificial conditions which no longer resemble the original PC situation. Studying such a confounded situation, however, can be interesting in its own right, since it resembles highly relevant everyday situations in which decisions have to be made and strategies have to be developed with regard to the proportion of congruent episodes. As an illustrative example, consider strategies of deception and faking in interactive sports (Güldenpenning et al., 2017). A basketball player may realize that trying to mislead an opponent by initiating a fake move with the head in a certain direction and then actually executing the opposite behavior with the body may be an efficient trick if applied rarely, but may lose its effect if applied on too many occasions (Güldenpenning et al., 2018, 2020). There are many possible explanations for this inverse relation between frequency and effectiveness of a deceptive move: For instance, after having been tricked, the opponent might exert cognitive control and focus more on the relevant information and thus become insensitive to the irrelevant movement; alternatively, the fake move might become associated not just with the erroneous response but also with the correct movement that should have been executed in hindsight, so that repeating the fake move will automatically trigger a retrieval of the efficient correct move (this is exactly what has been observed in two recent studies demonstrating goal-based retrieval of responses after errors, see Foerster et al., 2021; Parmar et al., 2022). Or it might result from associative contingency learning. If the effect of frequency of deceptions is driven by global adaptations of cognitive control or contingency learning, then the optimal solution would be to use deception only rarely; if local adaptations to the previous episode contribute most to the effect, then an alternating strategy would probably be the best solution; if the effect is driven by episodic retrieval of the last matching episode (i.e., the last encounter with this specific player), however, then it is not necessarily the immediately preceding event that is relevant, but the last episode in which a certain movement occurred, which can serve as a retrieval cue in the current situation, and the optimal strategy for the player would be to implement an alternating strategy regarding this cue.

## The present study

We aimed to systematically test the influence of episodic stimulus-based retrieval processes and to compare their contribution to the influence of global or local adjustments of control settings and contingency learning on PC effects in the Stroop task in a confounded setup, with a block-wise manipulation of the proportion of congruent trials. To achieve this aim, we analyzed the data of a standard congruency proportion paradigm with a multilevel modelling technique that allows us to represent different types of processes as independent predictors in a multi-level regression analysis, and to compare the predictive value of these predictors in a simultaneous regression analysis. We conducted a pre-registered, high-powered experiment with a standard block-wise PC manipulation in a color-identification task, using color as the relevant stimulus dimension and meaning of words as irrelevant stimulus dimension (i.e., distractors). Participants had to complete blocks of trials in which the proportion of congruent trials was either high or low. In a basic analysis, we established the standard PC effect: Responses on each trial were predicted by (a) the congruency of the color-word combination in a given trial, (b) by the proportion congruency in the respective block (representing global word-color contingencies and/or global control settings), and (c) by the interaction of these two factors, which indicates the PC effect. This interaction effect represents the influence of global (i.e., block-wise) word-color contingencies, and thus reflects predictions of the global accounts of the PC effect in terms of an adjustment of cognitive control settings (Botvinick et al., 2001) or contingency learning (Schmidt & Besner, 2008). We then included additional predictors into the regression equation that reflected each of the other proposed theoretical explanations of the PC effect. Specifically, in a second step we entered either (d) the congruency of the preceding trials and its interaction with the congruency of the current trial (congruency-sequence effect, CSE), reflecting the influence of local/trial-wise adjustments of cognitive control, (e) the response match between the last occurrence of the distractor and the current trial (reflecting episodic stimulus-based retrieval of responses), and (f) the congruency during the last occurrence of the distractor word and its interaction with congruency of the current trial (reflecting episodic retrieval of control states) as additional predictors, to test whether each of the proposed processes is able to explain the PC effect, as shown by a reduction or elimination of the interaction between congruency and block. Finally, we then compared the influence of these additional processes by entering multiple effects simultaneously, in order to see which of the effects dominates the other.

## Method

### Ethics vote, pre-registration, and open access

Ethical approval was granted by the Ethics Committee of the FSU Jena (FSV 20/005). Prior to data collection, the exact method, design, hypotheses, data preparation, and planned analyses were pre-registered online (https://aspredicted.org/3XX_38T).^1^ All data and analyses scripts are available at osf.io/vw6mz.

### Required sample size and a-priori power calculations

We ran a-priori power calculations to estimate required sample sizes with 1 – β = .80 and α = 0.05, for independent^2^ *t*-tests (one-tailed) and an effect size of d = .05 with G*Power 3.1 (Faul et al., 2007), which yielded a total of n = 102 (51 per group) to guarantee a sufficiently powered study (1 – β = .8).

#### Participants

In total, 109 participants were recruited online from a pool of students enrolled in different faculties at the FSU Jena (n = 36), and from Prolific Academic (n = 71). The final sample thus consisted of n = 109 participants (51 female, 56 male, 2 diverse; *M*_age_ = 23.4 years). All participants recruited via Prolific were pre-screened to be Native German speaking, aged between 18 and 30 years, using Windows 10 as an operating system and running the experiments on a notebook or desktop computer. The experiments had a duration of approximately 30 minutes and participants received either course credit (student sample) or £3.75 for taking part. All participants gave informed consent via key press prior to taking part in the studies.

#### Design

The experiment had a 2 (current trial: congruent vs. incongruent) × 2 (block: mostly congruent vs. mostly incongruent) × 2 (block order: mostly congruent block first vs. mostly incongruent block first) mixed factors design.^3^ In addition, each trial was coded according to (a) whether the immediately preceding trial was congruent or incongruent (congruency trial sequence), (b) whether the word of the current trial had been presented in a congruent or incongruent color during its last occurrence (retrieval of control states), and (c) whether the word of the current trial had been presented in a trial that required the same or a different color response during its last occurrence (stimulus-based response retrieval). Reaction times (RT) served as dependent variable of interest.

#### Materials and procedure

The experiment was programmed with E-Prime 3 and was converted for online data collection with E-Prime Go 1.0. At the start of each experiment, demographic information (gender, age, handedness) was collected, followed by the consent page. If participants consented to take part, instructions followed; otherwise, the study was terminated. Participants were informed that they would perform a color classification task.

The experimental stimuli for the color classification task were three color words (‘red’, ‘blue’, ‘green’) presented in either red, blue, or green color. Stimuli were presented against a black background. Each participant received two blocks of 450 trials each. In the mostly incongruent block, each of the nine possible word-color combinations were presented equally often (50 times), resulting in a congruency proportion of one third. In the mostly congruent block, each of the three congruent word-color combinations were presented 100 times (300 trials in total), while each of the six incongruent word-color combinations were presented 25 times (150 trials in total), resulting in a congruency proportion of two thirds. Within each block, the 450 trials were presented in an individually randomized sequence. Short breaks were inserted after 90 trials of the task; participants could continue the experiment by pressing the space key.

Before the experiment proper, participants familiarized themselves with the color classification task in a short practice block comprising 12 trials. The stimuli for the practice block were randomly chosen from the set of word-color combinations. The practice block had to be repeated until at least 10 out of the 12 responses were correct.

Each trial started with a white fixation cross that was presented for 250 ms in the center of the screen. The fixation cross was replaced by one of the colored word stimuli, which remained on screen until one of the three response keys was pressed on the keyboard of the computer. The neighboring ‘H’, ‘J’, and ‘K’ keys were used as response keys for the color classification task. Participants were instructed to use the index, middle, and ring finger of their dominant hand to respond, and not to move their fingers away from the response keys during the entire task. In case of an incorrect response, the colored word remained on the screen and the word ‘falsch’ (incorrect) was presented immediately below the color word until the correct response key had been pressed.

At the end of the experiment, we assessed participants’ contingency awareness by letting them indicate in which of the two blocks they saw more congruent word-color combinations.

### Data preparation

Prior to analyses, trials in which a color classification error occurred in the current (3.8%) or in the immediately preceding trial (3.6%) or during the most recent trial in which the word of the current trial had occurred (2.3%) were discarded. Trials in which a word occurred for the first time during the respective block and thus had no preceding episode that could have been retrieved were also discarded (0.7%). Also, responses faster than 250 ms or slower than 1.5 interquartile ranges above the 75^th^ percentile of the individual RT distribution were regarded as RT outliers (Tukey, 1977) and were excluded (3.8%). Data were analyzed with hierarchical multi-level linear regression analyses using single trials (nested within participants) as the unit of analysis. Analyses were conducted using the procedure *mixed* from SPSS 28 (IBM Inc., 2021). In these analyses, participants were treated as a level 2 factor to account for the statistical dependence between the trials of the same participant, with trials nested within participants.

## Results

### Block order effects

**Figure 2:**
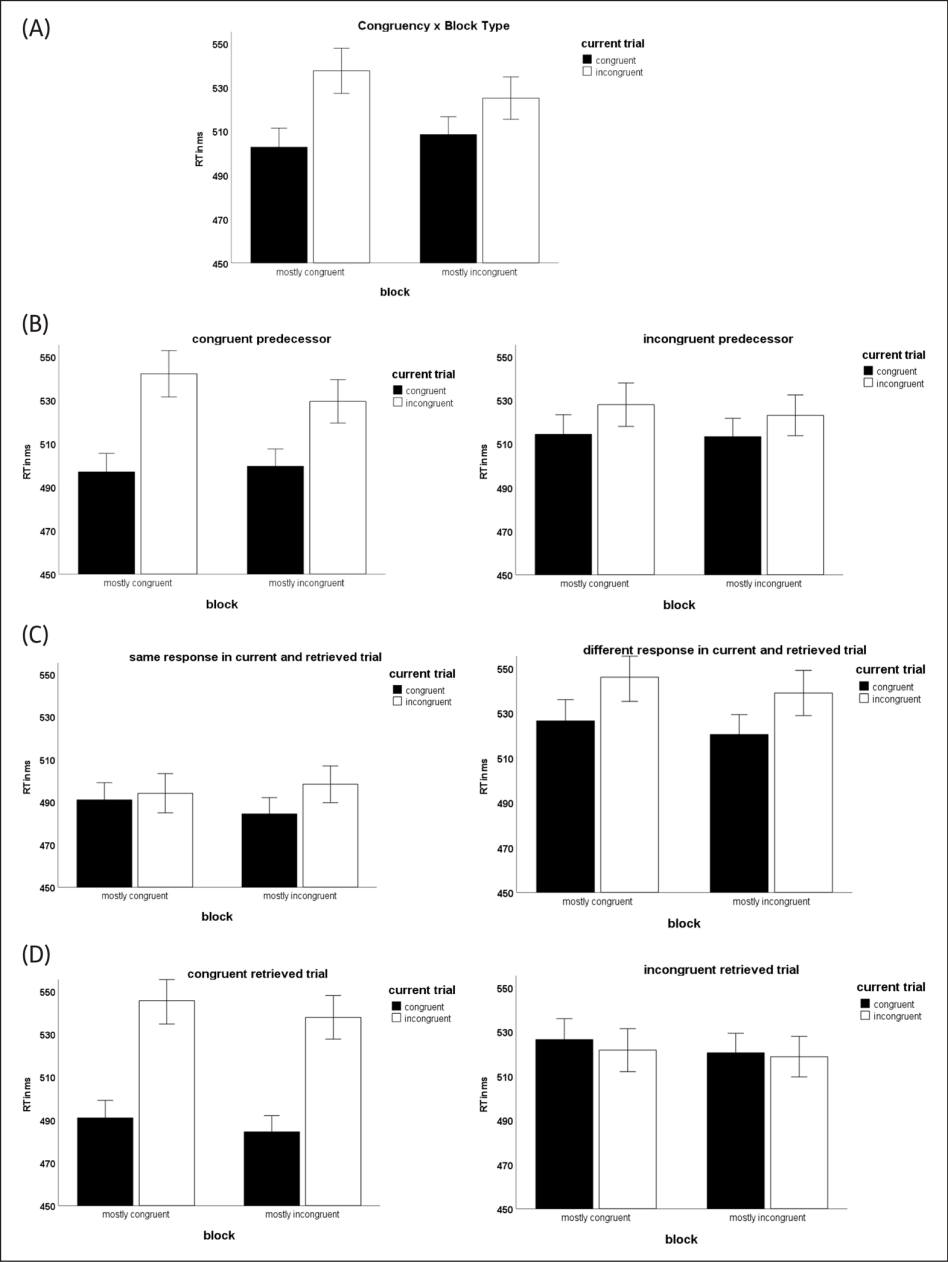
Pattern of proportion congruency (PC) effects under specific conditions corresponding to the models’ predictions (mean RTs; whiskers represent ±1 SE).

In a first analysis, we tested whether position (first vs. second block) modulated the PC effect, in order to decide whether the data should be analyzed in a within design (using data of both blocks for each participant) or in a between design (using only trials of the first block for each participant). RTs were regressed on the predictors current trial congruency (congruent = ½, incongruent = –½), block type (mostly congruent = ½, mostly incongruent = –½), position (first block = ½, second block = –½), and their interactions. The analysis revealed a robust effect for current trial congruency, *b* = –23.77, *t*(84,059.262) = –28.20, *p* < .001, that was modulated by the predicted interaction with block type, *b* = –13.50, *t*(84,059.136) = –8.01, *p* < .001, indicating that the congruency effect was stronger in the mostly congruent block. Importantly, however, this PC effect was further qualified by a significant three-way interaction with position, *b* = –8.48, *t*(84,059.262) = –2.52, *p* < .05, indicating that the PC effect was weaker for blocks that were presented at the second position. The interaction is driven by a difference in congruency effects for mostly congruent blocks, which were smaller after working through a mostly incongruent first block than when presented in the first block. This finding has been attributed to a decreased susceptibility to changes in congruency after a mostly incongruent block, maybe due to a stronger focus on just the relevant information (Abrahamse et al., 2013; but see Schmidt, 2016, for an explanation of the same effect in terms of the parallel episodic processing model [Schmidt et al., 2016]). The three-way interaction is indicative of transfer effects of the block that had been presented first on the subsequent second block. The second block thus cannot be considered a pure indicator of high vs. low congruency proportions, since participants were influenced by their previous experience from the first block, assimilating the second block to the first, which slightly dilutes the PC effect in the block that was presented last. We thus decided to use only the first block of each participant to get pure estimates of the PC effect for the following analyses.^4^ The pattern of means for the following analyses, corresponding to the models’ predictions, is shown in Figure 2.

### Proportion congruency (PC) effect

To establish the basic PC effect, we reran the previous analysis without the position factor, including only trials from the first block for each participant. The analysis yielded robust effects for current trial congruency, *b* = –25.58, *t*(42,390.416) = –21.55, *p* < .001, and for the interaction of current trial congruency by block type, *b* = –17.72, *t*(42,390.416) = –7.46, *p* < .001, indicating the standard PC effect with stronger congruency effects in the mostly congruent block (see Figure 2A).

### Congruency sequence effect (CSE)

To test whether the PC effect can be explained by a higher (lower) frequency of congruent preceding trials in the mostly congruent (incongruent) block, the congruence of the immediately preceding trials (preceding trial congruent = ½, preceding trial incongruent = –½), as well as the current trial congruency by previous trial congruency interaction were entered as additional predictors into the regression analysis (see Figure 2B for an illustration of the pattern of means). The analysis yielded significant effects for current trial congruency, *b* = –25.51, *t*(42,388.413) = –21.52, *p* < .001, for congruency of the previous trial, *b* = –3.87, *t*(42,388.219) = –3.28, *p* = .001, and for their interaction, *b* = –24.82, *t*(42,389.196) = –10.52, *p* < .001, indicating a larger congruency effect in the current trial after a preceding congruent trial that is in line with predictions of the adaptive cognitive control account. Importantly, however, despite controlling for the influence of congruency in the immediately preceding trial, the current trial congruency by block type interaction was still significant, *b* = –9.46, *t*(42,388.480) = –3.79, *p* < .001. Although the PC effect was smaller than in the original analysis, these findings indicate that it cannot be fully explained by the frequency of congruent and incongruent preceding trials.

### Episodic retrieval of responses

To test whether the PC effect can be explained by an episodic retrieval of mostly matching (mismatching) responses for congruent (incongruent) trials in the mostly congruent block, whereas congruent as well as incongruent trials should retrieve comparable percentages of matching (one third) and mismatching (two thirds) responses in the mostly mismatching block,^5^ we entered response relation between the current trial and the last occurrence of the word (same response during last occurrence = ½, different response during last occurrence = -½) as an additional predictor into the regression analysis (see Figure 2C for an illustration of the pattern of means). The analysis yielded significant effects for current trial congruency, *b* = –15.86, *t*(42,389.492) = –13.10, *p* < .001, and for response relation, *b* = –39.06, *t*(42,391.047) = –32.54, *p* < .001, indicating faster responses when the word had required the same color response during the last occurrence, which is in line with the prediction of the episodic response retrieval account. The current trial congruency by block type interaction was no longer significant in this analysis, *b* = 2.18, *t*(42,389.469) = 0.90, *p* = .37. General contingencies (represented by the block type factor) thus did not have an influence on the congruency effect after controlling for a trial-based retrieval of responses.

### Episodic retrieval of control states

To test whether the PC effect can be explained by a higher frequency of retrieving a relaxed control state (higher attentional weights for the word distractor) from the last trial in which the current word was shown in the mostly congruent (incongruent) block, the congruence during the last occurrence of the word (last occurrence congruent = ½, last occurrence incongruent = –½), as well as the current trial congruency by last occurrence trial congruency interaction were entered as additional predictors into the regression analysis (see Figure 2D for an illustration of the pattern of means). The analysis yielded significant effects for current trial congruency, *b* = –25.56, *t*(42,388.409) = –21.68, *p* < .001, for trial congruency during the last occurrence of the word, *b* = –7.84, *t*(42,388.216) = –6.68, *p* < .001, and for their interaction, *b* = –56.10, *t*(42,389.911) = –23.90, *p* < .001, indicating a larger congruency effect when the current word stimulus had been presented in a congruent color during its last occurrence, in line with predictions of the episodic retrieval of control states account. The current trial congruency by block type interaction was no longer significant in this analysis, *b* = 1.13, *t*(42,388.472) = 0.46, *p* = .65, indicating that the PC effect was eliminated after controlling for a trial-based retrieval of control states.

### Joint analysis

The previous analyses revealed that both the episodic retrieval of control states as well as the episodic response retrieval accounts can explain the PC effect, completely explaining and eliminating the effects of global (block-wise) cognitive control settings or contingencies. In order to decide which of the two retrieval processes actually is responsible for the effect, it is important to consider the influence of the two processes simultaneously, since the two retrieval processes share a large degree of overlap. We thus conducted another regression analysis, in which both the trial congruency during the last occurrence (and its interaction with congruency of the current trial), representing a retrieval of control states, as well as the response relation between the current trial and the last occurrence, representing response retrieval, were entered simultaneously as additional predictors into the model. This analysis revealed significant effects for current trial congruency, *b* = –14.74, *t*(42,387.698) = –11.51, *p* < .001, and for response relation, *b* = –43.58, *t*(42,388.971) = –21.10, *p* < .001. Importantly, the interaction effect of congruency during the last occurrence and congruency of the current trial, *b* = 9.40, *t*(42,388.651) = 2.42, *p* < .05, albeit significant, no longer played a role for the explanation of the PC effect, since the direction of the effect was now reversed, indicating slightly stronger congruency effects for trials in which the last occurrence of the word had been incongruent, which is inconsistent with the prediction of the retrieval of control states account. This small effect thus must be considered as a suppressor effect (i.e., it is used to bind irrelevant variance in other predictors), rather than representing an effect that reflects a retrieval of previous attentional control settings. As in the previous analyses, the current trial congruency by block type interaction was not significant, *b* = 1.32, *t*(42,387.468) = .53, *p* = .59, indicating that the block-based PC effect was fully explained via episodic response retrieval processes.

As an additional indicator of the contributions of different sets of predictors we computed the model fit for the full model (containing predictors coding both the episodic retrieval of responses and the episodic retrieval of control states) and for the models containing only one set of predictors (see Table 1).^6^ The analyses support the conclusions based on the significance tests for the multilevel analyses reported above: Including predictors coding an episodic retrieval of responses and an episodic retrieval of control states results in a substantial increase in model fit as indicated by lower values for the Bayes Information Criterion (BIC) and inverse log likelihood (2-LL) values for the full model compared with the simple model that includes only those predictors that code for the PC effect. Dropping the predictor coding an episodic retrieval of responses leads to a substantial decrease in model fit, indicating that this predictor is crucial for explaining the pattern of findings, whereas eliminating predictors coding an episodic retrieval of control states only leads to a negligible reduction in model fit, indicating that this process does not add anything substantial to the prediction of RTs over and above the episodic retrieval of control states.

**Table 1:** Fit indices (BIC: Bayes Information Criterion, Log likelihood: 2-LL) for different models predicting RTs on the basis of both episodic retrieval of responses and control states (full model), and from models in which one or both sets of predictors were dropped.


PREDICTORS^A^	BIC	2-LL

full model: C, B, C × B, RR, CR, C × CR	523,114,60	523,093.29

w/o retrieval of control states: C, B, C × B, RR	523,129.96	523,108.64

w/o retrieval of responses: C, B, C × B, CR, C × CR	523,560.78	523,539.46

simple model: C, B, C × B	524,177.80	524,156.49


^a^ C: congruency (current trial), B: block type (mostly congruent/incongruent), RR: response relation (between the current trial and the last occurrence of the distractor word), CR: congruency relation (between the current trial and the last occurrence of the distractor word).

## Discussion

PC effects were investigated in an experimental design with blocks of Stroop trials comprising either mostly congruent (2:1) or mostly incongruent (1:2) word-color combinations. Replicating previous findings (e.g., Logan & Zbrodoff, 1979), Stroop effects were much stronger in mostly congruent blocks, indicating a robust PC effect. The PC effect vanished after statistically controlling for either the congruency or the response relation of the last episode in which the word of the current trial had occurred. Apparently, PC effects in a blocked design in which multiple possible underlying processes compete with each other are not driven by global adjustments of cognitive control nor by word-color contingency learning, but were due to episodic retrieval processes.

In a mostly congruent (incongruent) block, retrieved episodes are mostly congruent (incongruent); re-activating the cognitive control state of the most recent episode in which the current word had been presented should thus lead to a stronger (weaker) influence of the words on responding in the current trial, producing large vs. small Stroop effects in the respective blocks. Similarly, retrieving mostly congruent episodes in a mostly congruent block leads to a re-activation of mostly matching (mismatching) responses for congruent (incongruent) trials, and should produce a large Stroop effect, whereas retrieving mostly incongruent episodes in a mostly incongruent block leads to a re-activation of similar proportions of matching/mismatching responses for both congruent and incongruent trials, which should produce a smaller Stroop effect.

Having both types of episodic retrieval processes compete against in each other by entering both simultaneously as predictors into the regression analysis revealed that the PC effect was driven entirely by episodic response retrieval. Episodic retrieval of control states no longer had any influence on responding after controlling for response retrieval, and similar conclusions were suggested by comparing changes in model fit after dropping predictors relating to an episodic retrieval of control states or to an episodic retrieval of responses from the full model. These findings indicate that the previously obtained effect of the respective interaction of congruency of the last occurrence and current congruency was due to a spurious relation with response retrieval processes. If a congruent last episode is retrieved, both accounts make exactly the same prediction: Retrieving a relaxed control state should lead to strong facilitation/interference for congruent/incongruent trials according to the episodic retrieval of control states. At the same time, a previous congruent episode would also share the response with a current congruent episode, and would have a different response if the current trial is incongruent. If an incongruent last episode is retrieved, however, predictions differ, which allows for a competition of the different predictions: Retrieving a focused state of cognitive control should weaken facilitation/interference for congruent/incongruent current trials according to the retrieval of control states account. While a previous incongruent episode would always have a response that differs from a current congruent trial (interference), it will either match or mismatch with the response of a current incongruent trial, depending on which of the two possible incongruent colors is presented. Eliminating this confound by statistically controlling for the influence of response retrieval also eliminated the effect of previous control states on the PC effect.

At this point, it should be noted that the current design does not allow for a fully orthogonal variation of the two types of retrieval processes. Due to the nested structure of the design (with congruent-congruent distractor repetition sequences from trial *n-i* to trial *n* implying a response match between these trials), no interaction effects of the two factors can be tested, which might result in biased estimates for the main effects of the two factors that could “steal” variance from interaction effects that could not be included into the model, and which might bias the estimation of the contribution of the main effects of the respective factors (Schmidt et al., 2014). To get rid of these limitations, an independent experimental manipulation of congruency relation and response relation is necessary, which can be achieved by making episodic retrieval dependent on a third dimension of the stimuli that are presented in the Stroop task (e.g., by introducing color-neutral carrier words that drive episodic retrieval, and by manipulating color congruency by presenting the task-relevant color on the carrier word, and the distractor colors as flanker stimuli).^7^

It is also important to note that more specific forms of adjusting cognitive control on a trial-based basis as reflected in the CSE (Gratton et al., 1992), or learning of item-specific contingencies also cannot explain the current findings, which is in line with previous studies suggesting that the CSE and PC effects reflect independent mechanisms (e.g., De Pisapia & Braver, 2006; Funes et al., 2010; Torres-Quesada et al., 2013; but see Aben et al., 2017). First, our data demonstrate that controlling for congruency sequence effects (congruent vs. incongruent immediately preceding trial) reduced but did not eliminate the block-based PC effect. Local adjustments of cognitive control-settings on a trial-to-trial basis thus provides no convincing explanation for the PC effect. Similarly, a learning of item-specific contingencies cannot explain why the retrieval of an infrequent word-color combination during its last occurrence reverses the effect of the overall contingency.

### Limitations, implications, and conclusion

Our study employed a simple, block-wise proportion congruency manipulation. This type of design does not allow us to empirically distinguish effects of global adjustments in cognitive control (or of a cumulative version of context-dependent retrieval of control states) from effects of contingency learning since both accounts make identical predictions under these conditions. We feel justified in using such a simple design, since this contrast was not the main focus of interest of our study. Instead, we wanted to investigate whether episodic retrieval accounts could explain the PC effect, ruling out explanations in terms of global adaptions in attentional control settings and contingency learning alike. The results of our study clearly demonstrate that in a standard blocked design, episodic retrieval of the most recent episode actually is the driving force that explains block-level congruency effects in such a confounded design.

Of course, our conclusions cannot and should not be generalized to other, more complicated or sophisticated experimental designs and manipulations. It is perfectly possible that more global effects of cognitive control adjustments or contingency learning may explain PC effects that emerge under different, experimentally controlled conditions. At least, however, our findings suggest that episodic retrieval processes should be considered as an important competing predictor of PC effects under any conditions – just controlling for or eliminating immediate feature repetitions (identical vs. partial repetitions) from trial n-1 to trial n is not sufficient to rule out the influence of episodic retrieval processes, since episodic retrieval processes are not restricted to immediate sequences of trials but may be effective across multiple intervening trials (e.g., Giesen et al., 2020; Schmidt et al., 2020), nor does a control of partial and exact repetitions fully account for retrieval effects since (a) partial repetitions do not distinguish between a condition in which the distractor is repeated that retrieves a mismatching response or target (leading to interference), and a condition in which the distractor changes so that no retrieval (and no interference) occurs. Furthermore, (b) an analysis of feature repetitions cannot account for the finding that distractor-based retrieval produces strong facilitation even in situations where the target feature did not repeat (i.e., when a many-to-one mapping was used to disentangle effects of repetitions from retrieval proper; Frings et al., 2007; Giesen et al., 2012), demonstrating facilitation under conditions of partial feature overlap. Finally, even exact feature repetitions can lead to interference, when the response assignment changes between successive trials (Rothermund et al., 2005), indicating again that it is response retrieval rather than (exact vs. partial) feature repetition what drives the effects.^8^

We recommend the use of hierarchical multilevel analyses as a versatile tool to simultaneously investigate the influence of competing processes and to estimate the unique contribution of these processes to the explanation of global PC effects under confounded conditions. The present study shows that this method provides interesting insights into the processes underlying global PC effects in a confounded design and that is also superior to merely eliminating immediate stimulus repetitions from the data set, which does not eliminate episodic retrieval of more distant last occurrences of the current trial stimulus.

Our findings provide further evidence for the strong influence of episodic retrieval processes on responding in simple response time paradigms. In line with recent computational models of episodic retrieval processes (Schmidt et al., 2016), the very last episode in which a stimulus occurred apparently has a huge influence on these retrieval processes, attesting to the power of the recently proposed “law of recency” (Giesen et al., 2020).

## Data accessibility statement

All data and analyses scripts are available at osf.io/vw6mz.

## Footnotes

During pre-registration, we mainly focused on contrasting the episodic response retrieval account with global explanatory accounts (contingency learning, cognitive control) of the PC effect. We were not yet considering an episodic retrieval of control states account, but later realized that our data provide an ideal test bed to also compare different versions of the episodic retrieval account (response retrieval vs. retrieval of control states). Although analyses relating to this later account were not pre-registered, we decided to include them into the analyses to give a more comprehensive account of which is the decisive process underlying the PC effect. Another deviation from the pre-registration is that we decided not to include the distance to the last occurrence of the distractor as an additional factor into the regression equation, although we pre-registered such an analysis. The reason for our decision was not to make the analyses unnecessarily complex, and not to favor the retrieval effects over the CSE effect by adding more predictors (distance, distance x last occurrence) into these models. Adding the distance factor and its interactions, however, does not change the results and leads to the same conclusions.

Although we used a within design with counterbalanced order of mostly congruent and mostly incongruent blocks, we decided to calculate the required sample size on the basis of a between design, since we wanted to make sure that we could analyze the data reliably also in a between design in case of a cross-level interaction between the congruency proportion effect and the balancing factor (sequence), indicating the PC effect would differ for the first and second position due to transfer effects.

Block order was determined randomly with 51 (58) participants receiving the mostly congruent (incongruent) block first.

The decision to make the type of analysis in a between vs. within design dependent on the existence or non-existence of a significant modulation of the PC effect by position followed from the rationale that was pre-registered for this study. Although significant, the size of the three-way interaction was comparatively small in relation to the size of the PC effect. We thus decided to run additional analyses with the full data set in order to check the robustness of our findings and conclusions, which yielded an identical pattern of effects for all subsequent analyses. Results of these analyses can be obtained from the first author upon request.

In mostly incongruent blocks, each word was presented with the same probability in each of the three colors. Both congruent and incongruent trials thus had an identical probability of retrieving a previous trial with the same/matching (1/3) or a different/mismatching (2/3) color response.

We thank the editor, Prof. Dr. Andrea Kiesel, for suggesting these additional analyses, based on a recent paper by Spitzer et al. (2022). These analyses of model fit were not preregistered.

We want to thank Prof. Dr. David Dignath and Moritz Schiltenwolf for making us aware of the problems that are inherent in the nested structure of our design, and for suggesting an ingenious idea how to experimentally get rid of this limitation. Following a recommendation from Prof. Dr. James Schmidt, we conducted another analysis (not pre-registered) in which only trials with an incongruent color-word combination were analyzed, since only for those do the two episodic retrieval accounts make different predictions (cf. Schmidt, 2013b; Schmidt & Lemercier, 2019). This analysis revealed a strongly significant effect for episodic response retrieval (*t* < 20), no effect for episodic retrieval of control states (*t* < 1), and no block effect (*t* < 1), which further corroborated the conclusions of the previously reported analyses.

Based on suggestions of an anonymous reviewer, we also conducted an additional analysis (not pre-registered) in which we tested whether immediate full and partial feature repetitions (coded by two separate dummy predictors contrasting exact repetitions and partial repetitions against complete alternations, respectively) could explain the PC effect. These analyses revealed that controlling for immediate repetitions did not eliminate the PC effect (the block type × congruency interaction is still highly significant after controlling for immediate complete and partial repetitions, *t* > 5).
